# Inferring hidden causal relations between pathway members using reduced Google matrix of directed biological networks

**DOI:** 10.1371/journal.pone.0190812

**Published:** 2018-01-25

**Authors:** José Lages, Dima L. Shepelyansky, Andrei Zinovyev

**Affiliations:** 1 Institut UTINAM, Observatoire des Sciences de l’Univers THETA, CNRS, Université de Franche-Comté, 25030 Besançon, France; 2 Laboratoire de Physique Théorique du CNRS, IRSAMC, Université de Toulouse, CNRS, UPS, 31062 Toulouse, France; 3 Institut Curie, PSL Research University, Mines Paris Tech, Inserm, U900, F-75005, Paris, France; 4 Laboratory of advanced methods for high-dimensional data analysis, Lobachevsky University, Nizhni Novgorod, Russia; Friedrich-Alexander-Universitat Erlangen-Nurnberg, GERMANY

## Abstract

Signaling pathways represent parts of the global biological molecular network which connects them into a seamless whole through complex direct and indirect (hidden) crosstalk whose structure can change during development or in pathological conditions. We suggest a novel methodology, called Googlomics, for the structural analysis of directed biological networks using spectral analysis of their Google matrices, using parallels with quantum scattering theory, developed for nuclear and mesoscopic physics and quantum chaos. We introduce analytical “reduced Google matrix” method for the analysis of biological network structure. The method allows inferring hidden causal relations between the members of a signaling pathway or a functionally related group of genes. We investigate how the structure of hidden causal relations can be reprogrammed as a result of changes in the transcriptional network layer during cancerogenesis. The suggested Googlomics approach rigorously characterizes complex systemic changes in the wiring of large causal biological networks in a computationally efficient way.

## Introduction

The network biology point of view on signaling pathway as a part of the complex integrated molecular machinery consists in considering it as a subnetwork embedded into a global molecular network. As a consequence, all properties of the pathway functioning depend on the network context to which it remains connected. Considering only the set of direct causal relations between pathway members (as is frequently the case) neglects the indirect effect of the global biological network changes which may significantly re-wire the pathway topology by introducing implicit (hidden) causal relations. Characterizing the global network structure influence on the local network properties and dynamics remains one of the major challenges of systems and network biology [1]. A number of empirical and pragmatic approaches have been suggested recently to address this question [2–6].

The global biological molecular network is composed of several large parts, having different nature of interactions, time scales and roles in cellular life. Thus, signaling network has a role of propagating the signals received from cellular environment to nucleus via complex cascades mainly based on interactions between proteins, such as kinases and their targets, and involving numerous post-translational protein modifications such as phosphorylation, acetylation, ubiquitination. By contrast, the transcriptional network has a primary role of global and long-term cell reprogramming assuring robust cell fate decisions. Connections in the transcriptional network are based on protein-DNA interactions between transcription factors and their target genes via binding to promoter and enhancer DNA sequences. Signaling and transcriptional networks are strongly intertwined in the cell, providing multiple feedbacks from one to an other.

Availability of computational reconstructions of all types of molecular networks are critical for understanding the connection between genotype and phenotype [1]. Global reconstructions of the directed causal signaling network structure have appeared only recently in the form of comprehensive molecular interaction databases such as SIGNOR [7], SignaLink [8], where the pair-wise relations between molecules are oriented. Large-scale cell type-specific reconstructions of transcriptional causal networks have become possible thanks to appearance of Chip-Seq technology [9, 10] or advances in computational methodology of transcription factor binding site predictions combined with the data on chromatin accessibility [11].

In this study, by transcriptional network we understand a directed graph representing relations between two types of nodes: transcriptional factors and their targets (among which there can be transcriptional factors as well). By signaling network we mean a graph of regulatory interactions, where the nodes represent proteins and the edges represent directed causal influences of activity of one protein onto the activity of another one, involving principally protein-protein interactions. We assume that merging these two graph types represent a combined hybrid network describing co-existence of relatively fast signaling and relatively slow transcriptional feedback, in turn affecting the signaling.

The main question we want to answer is to quantify the effect of one protein onto an other one, taking into account the global network structure. In previous works, quantification of indirect interactions (sometimes called influences) between pathway members mainly exploited the calculation of shortest or second shortest paths (the paths that become shortest after removal of an edge in a shortest path), followed by quantification of the balance between negative and positive path signs [6, 12]. The limitation of such approaches is, however, in that they do not take into account the whole complexity of the global network structure: multiple dense causal connections between two nodes might be more important than a single shortest path connecting them.

Global changes in the structure of the global network might effectively re-wire a signaling pathway even if its direct interactions depend weakly on the biological context. For example, the functioning of a signaling pathway must be heavily affected by the structure of transcriptional feedbacks indirectly re-wiring the pathway structure by gross effect of implicit (hidden) causal relations. Therefore, changing the transcriptional layer in the global network effectively rewires many signaling pathways even without affecting the structure of direct connections between its members. It creates a need in developing an efficient and rigorous mathematical formalisms allowing quantifying such a phenomenon.

Google matrix formalism formulated by Sergei Brin and Lawrence Page is the mathematical basis of Google Search Engine [13]. It assumes the following model of a hypothetical user web-surfing behaviour. The user starts to navigate in Internet from a random page, follows the hyperlinks by random clicking for a certain random time, after which the user jumps (teleports) to a randomly chosen page, and continues. Google matrix is a stochastic matrix describing this random process, and the PageRank value of a page is defined as a probability of visiting this page via the random surfing in infinite time. Evidently, pages with many incoming links have more chances to obtain a PageRank closer to the top. One can think of reversing the random web-surfing process by changing the orientation of all hyperlinks (a user is lead from the current page to one of the pages pointing to it). The reversed process is described by another stochastic matrix, which defines the CheiRank probability introduced in [14]. Oppositely to PageRank, CheiRank is closer to top for the pages with many outgoing hyperlinks.

Following similar ideas, we assess the characteristics of signal propagation through a pathway by considering the stochastic Markov process of random walk with uniform non-zero restart (teleportation) probability along oriented edges of the graph representing the global biological network. This process is described by Google matrix (see Materials and Methods section), and its stationary state defines PageRank centrality measure of the graph nodes. More complete and subtle description of the process can be obtained by looking at the complete (complex) spectrum of the Google matrix, which might reflect complex non-stationary properties of the random walk. For example, a set of close eigenvalues of the Google matrix on the complex plane can define weak communities in the graph where the signaling flow (random walk) can be “trapped” for a finite time.

The Google matrix approach or related random walk-based approaches is now a well established efficient tool for analysis of various complex directed networks including the World Wide Web [13], citation network of Physical Review [15], rating of global importance of scientific journals [16], world trade commercial networks and many other networks [17, 18]. A general review of the methods for the analysis of directed network structure can be found in [19].

To our knowledge, the Google matrix approach or related ideas have been applied before in systems biology mostly to undirected networks, with few exceptions (e.g., [20]), in order to find activated network modules or to “smooth” high-throughput data [21–23], to establish connection of genes to diseases [24, 25], to improve interpretability of genome-wide analyses [26–28] and to compute network-based cancer biomarkers [29, 30]. PageRank approach has been used to quantify the functional proximity in undirected protein-protein interactions networks [31]. A comprehensive recent review on the application of random walk with restart approach to biological networks can be found in [32]. Comparison of the Google matrix algorithm with other approaches for quantifying centrality (e.g. in-degree/out-degree distributions, betweenness centrality, closeness centrality) are discussed in detail in [33–36].

In order to quantify indirect hidden causal relations between members of a pathway, here we exploit the analytical formalism of reduced Google matrix developed recently in [37] and applied in the context of political studies [38]. If we continue to use the Internet as a source of intuition, the reduced Google matrix describes random web-surfing of a user, but assuming that we are able to detect the user presence only at a restricted set of pages *R*. Direct hyperlinks between pages from this set are described by the corresponding global Google matrix. We want to predict what will be the probability of observing a user on a page *b* ∈ *R* when he leaves *R* from the page *a* ∈ *R* to any page *c* ∉ *R*. This computed probabilities of indirect transitions will be denoted as *G*_*qr*_ further in the text. The reduced Google matrix describes both direct *G*_*rr*_ and indirect *G*_*qr*_ transitions. Its computation is based on decomposing the global Google matrix into the parts describing the set *R* itself and the transitions in the rest of the network (see Methods section).

The efficiency of the reduced Google matrix analysis has been demonstarted in the context of political sciences [38, 39] on the basis of networks of Wikipedia in different language editions. The strong new features of the reduced Google matrix is that it constructs a Google matrix for a certain subset of nodes of relatively small size *N*_*R*_ (in which one is interested in) being much smaller than the huge global network with millions of nodes (as for Wikipedia, see [38, 39]). At the same time all indirect interactions between the selected *N*_*R*_ nodes, taking place via the huge global network, are taken into account as explained in [38, 39] and the Methods section below. In this sense the reduced Google matrix method is rather unique since all direct and indirect interactions of the global network are taken into account and the PageRank probabilities of the global network are preserved for the selected *N*_*R*_ nodes (up to a normalization constant). The formalism of reduced Google matrix is applied for biological networks in this paper for the first time.

We describe the details of the Google matrix and the reduced Google matrix methodology in the “Methods” section. The application of the methodology to several large regulatory networks is documented in the “Results” section. Using the suggested approach, we quantify the effect of the changes in the structure of transcriptional network as a result of oncogenic events during chronic myelogenous leukemia onto re-wiring connections between proteins in several cancer-related groups of genes. We conclude that the method is able to infer the missing indirect causal relations between the members of a pathway and can detect events of implicit network re-wiring during cancerogenesis.

## Results

### Used networks and case study description

In order to illustrate the application of Google matrix approach to studying oncogenic changes in the global and local network structures, we constructed two large directed networks describing global signaling in a leukemia cancer cell line K562 compared to a healthy cell line GM12878 derived from normal B-lymphocytes. The transcriptional networks of these two cell lines have been previously characterized [10], using systematic Chip-Seq experiments on a number of transcription factors whose activity is detected in a given cell line. Transcriptional networks for GM12878 and K562 cells have been previously analyzed in order to quantify and compare their structural properties related to buffering and robustness [40]. It was shown that the wiring of the transcriptional network in cancer leads to significant changes in the number of structural patterns leading to degrading the network robustness properties. However, this analysis was limited to the structure of the transcriptional networks only, the signaling part of the network was completely neglected. In order to deal with combined signaling+transcriptional networks, we merged each of the transcriptional network to the global reconstruction of signaling taken from the SIGNOR database [7] (version from February 2016).

**Fig 1:**
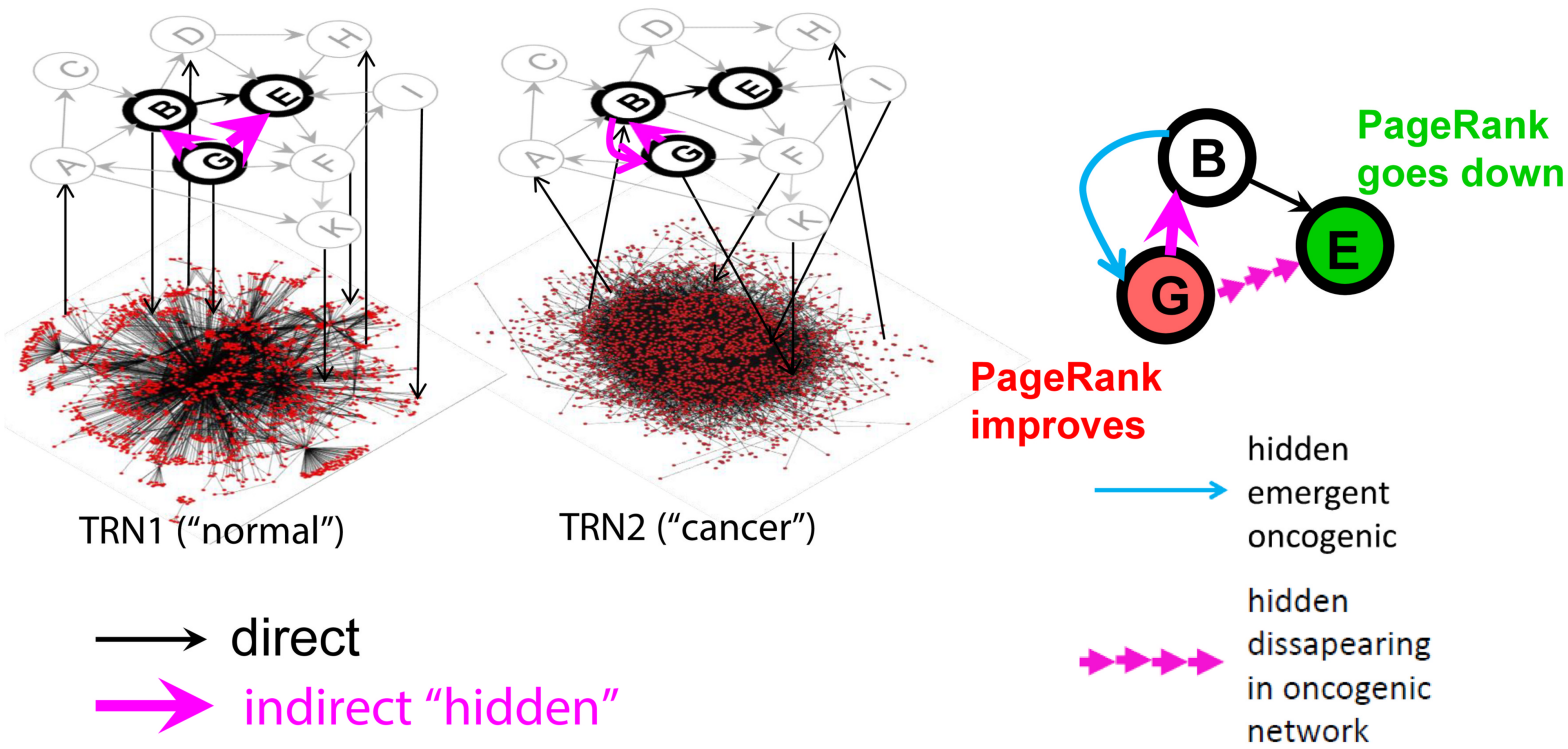
Using reduced Google matrix approach for inferring hidden causal relations in signaling pathways. Here the structure of the context-dependent global regulatory network is symbolically shown as consisting of two layers: the upper (nodes A-K) is the global signaling network whose structure does not depend on the context and the lower is a symbolic view of the contextual transcriptional regulatory network (TRN) whose structure can change between a “normal” and a “cancer” cell. Thick node borders denote a pathway embedded into the global signaling network. Black arrows denote direct physical interactions. Pink arrows denote inferred hidden directed regulations through the global regulatory network (both layers). In the final representation of the pathway (on the right), one can show those hidden regulations which emerge or disappear due to the changes in the TRN structure. Also, the color of the pathway nodes can show the direction of PageRank change: green corresponds to the PageRank decreased in the cancer network while red corresponds to the opposite.

As a simplifying modeling hypothesis, we assume that the structure of the global signaling network depends less on the biological context than the structure of the transcriptional regulation layer (Fig 1). Although this is a relatively strong hypothesis, it can be qualitatively justified here by the following considerations. First, in the textbooks on the biology of cancer, it is frequently assumed that rapid and coarse-grained evolution taking place during cancerogenesis does not have time to define new interactions between protein domains [41]. Therefore, we can assume that “re-wiring” of protein-protein interaction network structure is relatively limited during cancer progression. By contrast, there are multiple epigenetic mechanisms strongly affecting the transcriptional network structure. Second, from pragmatic considerations we have yet very rarely access to the global reconstructions of context-specific protein-protein interaction networks, while the context- and disease-specific transcriptional network reconstructions become abundant [42]. Third, in this study, we specifically focus on the role of indirect “rewiring” of protein-protein interaction modules, through the changes in the transcriptional network. Therefore, estimating the amplitude of this effect is easier if we assume that only the transcriptional network structure changes.

### Biological interpretation of PageRank and CheiRank centrality measures and their changes in cancer

#### Distribution of proteins on PageRank vs CheiRank plane

**Fig 2:**
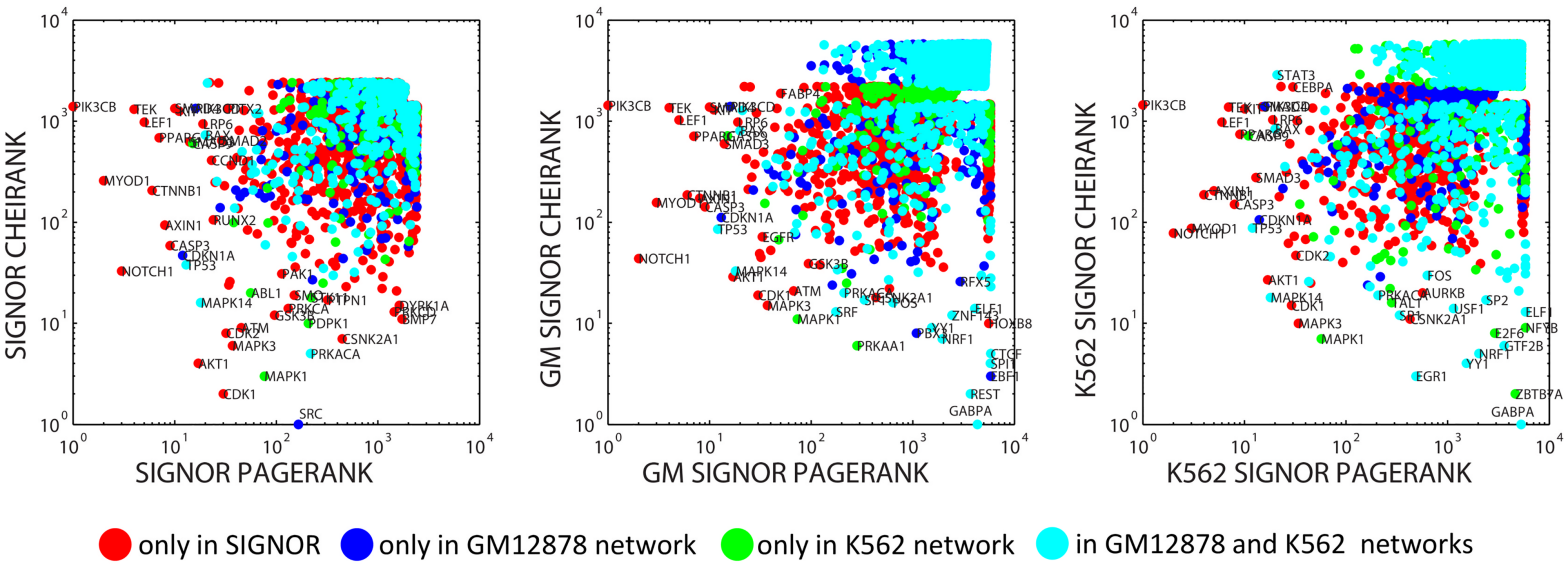
Distribution of proteins on PageRank x CheiRank plane for three networks (SIGNOR—signaling network, GM network is a merge of SIGNOR and GM12878 normal blood cell transcriptional network, K562 network is a merge of SIGNOR and K562 leukemia cancer cell transcriptional network). The colors signifies to which network a protein originally belongs. Red color signifies proteins which are present only in SIGNOR network and not present in any of the two transcriptional networks. Dark blue color signifies proteins which are present in GM12878 transcriptional network and not present in K562 transcriptional network. Green color signifies proteins which are present in K562 transcriptional network and not present in GM12878 transcriptional network. Cyan color signifies proteins which are present in both K562 and GM12878 transcriptional networks.

For the three directed biological networks described above (SIGNOR alone and two merged signaling+transcriptional regulatory networks), we applied the Google matrix methodology as described in Materials and Methods section, and determined the values of PageRank and CheiRank for all proteins. The distribution of proteins in PageRank vs CheiRank plane is shown in Fig 2. This figure shows that in the case of these networks PageRank and CheiRank measures are not correlated which reflects quite distinct biological role of proteins with many incoming and with many outgoing directed interactions.

It can be easily demonstrated (data not shown) that most of the proteins simultaneously having high values of PageRank and CheiRank (such as AKT1, NOTCH1, CTNNB1, TP53, CDKN1A, ATM, MAPK3, CDK1, EGFR) play an important role in cancer biology. This is, however, a rather trivial observation having in mind that it is known that hubs of the protein interaction networks frequently correspond to cancer-related genes [1, 43].

One can also observe that adding a transcriptional network to a signaling network (SIGNOR) significantly changes the top ranked proteins for CheiRank but not for PageRank. This is consistent with the fact that the transcriptional networks are characterized by fan-like structures in which a transcription factor can regulate many (hundreds) of proteins, while the cases when a protein is regulated by so many upstream regulator are relatively rare.

Overall, the general shape of the distribution of proteins in the “normal” GM (Fig 2, middle) and “cancer” K562 (Fig 2, right) networks is similar. Nevertheless, there exist several noticeable differences between the two. For example, it can be seen that the top Chei-ranked proteins are not the same in these two networks. There is a region in the PageRank vs CheiRank plane for GM network (Fig 2, middle) occupied by proteins which are present in SIGNOR but not in GM12878 transcriptional network (cluster of green color points for CheiRank and PageRank around 1000). Vice versa, there is a region in K562 network (Fig 2, right) occupied by proteins which are present in SIGNOR but not in K562 transcriptional network (cluster of dark blue points). This observation underlines the fact that both the composition and the wiring topology of “normal” and “cancer” networks have important differences.

#### Detecting “creative protein elements” by comparing PageRank and CheiRank to simple connectivity degree

**Fig 3:**
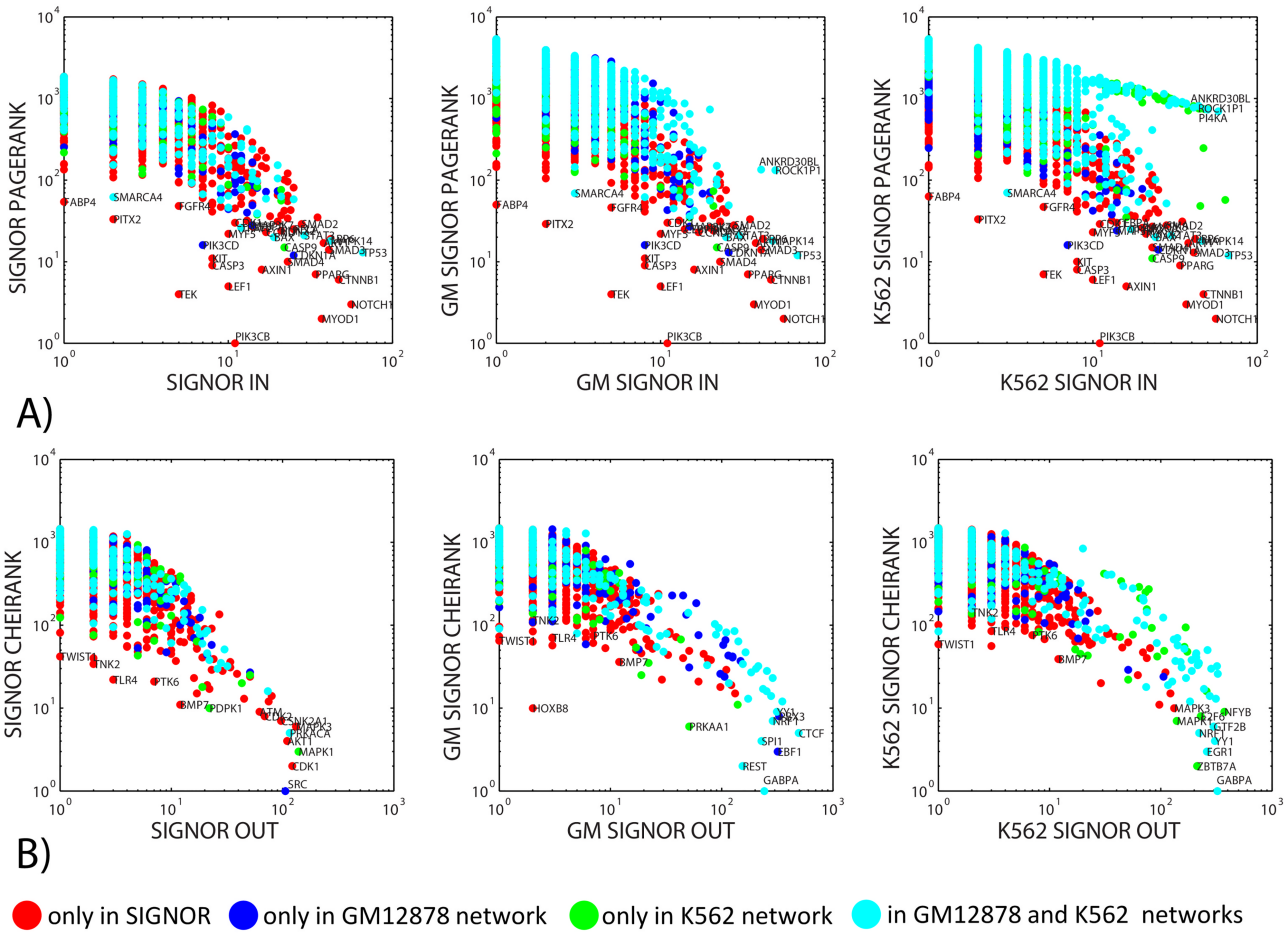
Dependence of PageRank (A) and CheiRank (B) on the in-degree and out-degree correspondingly, in three networks. The coloring of nodes corresponds to the description provided in the caption of the Fig 2.

A general statement on PageRank and CheiRank consists in that on average they are correlated to in-degree and out-degree of a node [44]. However, this correlation is not perfect as it can be seen in Fig 3. Some proteins significantly deviate from the general dependence trend, so it is interesting to consider what kind of non-local network topologies give unexpectedly high PageRank or CheiRank values despite relatively low connectivity degree. This deviation can be scored by a simple product of the rank and the corresponding degree, i.e., *CE*_*in*_ = *K* ⋅ (*InDegree* + 1), *CE*_*out*_ = K* ⋅ (*OutDegree* + 1).

The notion of “creative elements” in the context of biological networks is discussed in [2] as such proteins that are not hubs of the networks themselves but can provide important (and, frequently, transient) connections between network hubs. Also, sometimes, together with hubs, proteins playing the role of “connectors” are discussed as those proteins having high centrality but not high connectivity measures [1].

**Fig 4:**
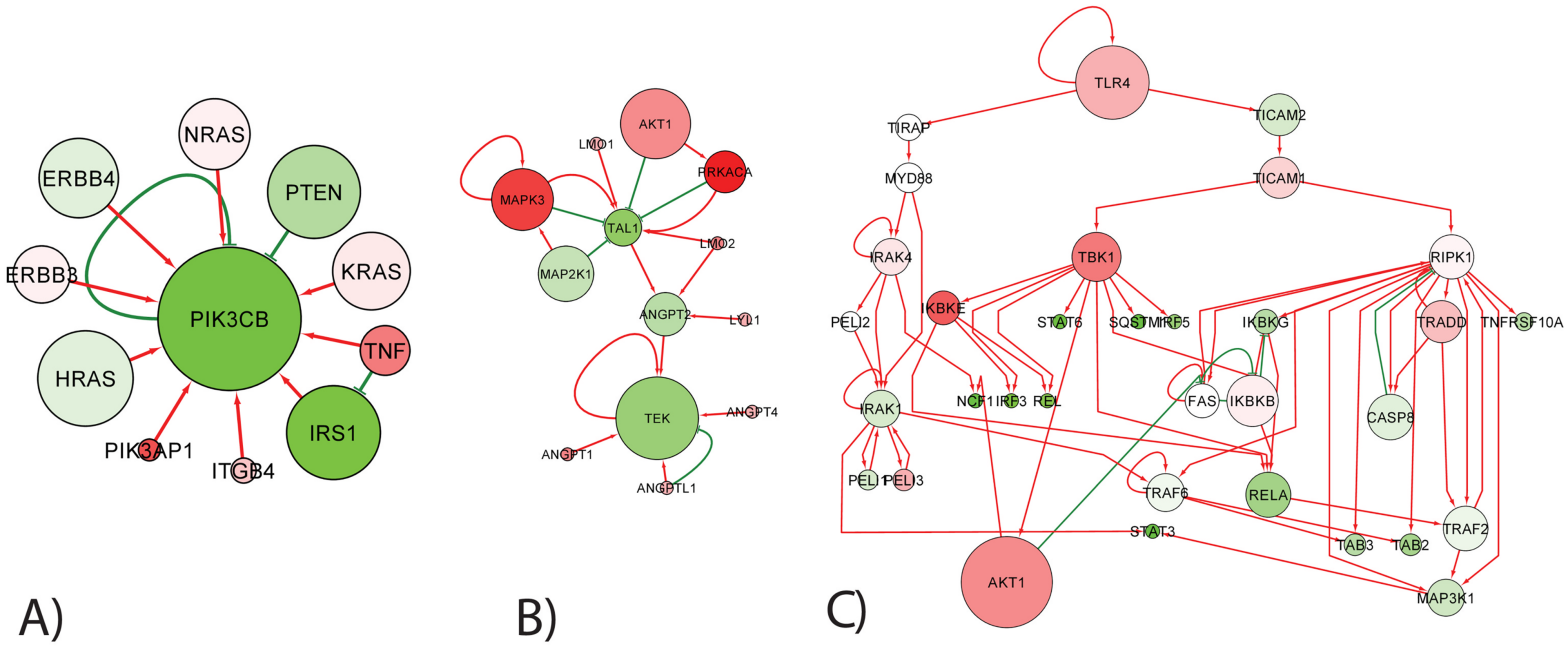
Several examples of network topologies, illustrating emergence of “creative elements”. Here creative elements are defined as proteins whose PageRank or CheiRank can not be simply explained by the number of incoming or outgoing connections correspondingly. A and B) The size of the node is proportional to its PageRank value. C) The size of the protein is proportional to its CheiRank value. The color of the proteins in all three panels reflects the ratio between the number of incoming and outgoing edges. If the color is greenish then the protein is “importer” receiving more incoming regulations than having out-going edges. The reddish proteins are “exporters” regulating more proteins downstream than the number of incoming regulations.

We suggest that the proteins significantly deviating from the the general trend between a Google matrix-based rank and the corresponding connectivity degree are potential candidates for the role of “creative elements” and connectors in the signaling and transcriptional networks. Several examples of such proteins and the local topologies explaining the deviation from the trend are provided in Fig 4.

For example, the top ranked in PageRank protein in all three networks is PIK3CB (look at Fig 2) having relatively low in-degree (10), which makes it to significantly deviate from the general trend (Fig 4A). PIK3CB gene encodes a catalytic subunit of the kinase PI3K, a key kinase involved in multiple cell signaling cascades. PIK3CB gene is frequently mutated or amplified in several cancer types (such as lung squamous cell carcinoma where its rate of mutations can be as high as 18%), which causes abnormalities in cell survival signaling. From the network point of view, the high value of PageRank is explained by the fact that, accordingly to SIGNOR, PIK3CB is regulated by several highly connected proteins which have predominantly incoming edges (PTEN, ERBB3, ERBB4, HRAS, NRAS, KRAS, IRS1).

An other example of such a protein is TEK which is ranked #4 by PageRank in SIGNOR network, having only 5 incoming edges. From Fig 4B one can see that TEK is a sink of the cascade TAL1→ANGPT2→TEK, which progressively collects incoming regulations, starting from the top connected hubs such as AKT1, MAPK3, PRKACA. The biological function of the TEK protein is quite unique: this is a receptor tyrosine kinase which has several immunoglobulin-like domains, three epidermal growth factor domains and three fibronectin type III repeats in the extracellular part. This makes this protein potential regulator of multiple cellular functions such as angiogenesis, endothelial cell survival, proliferation, migration, adhesion and cell spreading, reorganization of the actin cytoskeleton, and also maintenance of vascular quiescence. Such rich functional cross-talk allows suggesting TEK as a creative element in the global cell signaling network.

Our final example of deviation from the main CheiRank vs out-degree trend is TLR4 protein, which is ranked #22 by CheiRank in SIGNOR having only 2 out-going regulation and 1 self-interaction. From the Google matrix-based network analysis this can be explained by the fact that TLR4 triggers several cascades affecting several major downstream regulators that have a large number of out-going edges such as AKT1. Indeed, the biological function of TLR4 (toll-like receptor 4) is activating the innate immune system which requires triggering many important cellular cascades (such as NFkB signaling), regulating a large number of cellular processes.

Other observations from Fig 3 underlies some particular features and differences between “normal” and “cancer” networks. For example, one can notice in Fig 4A, right that in the cancer network there is a relatively large number of proteins with high number of incoming transcriptional regulations but not ranked well by PageRank. This feature is almost absent in the normal GM-SIGNOR network (Fig 4A, middle).

#### Biological meaning of PageRank and CheiRank changes in cancer

Having seen the differences in the distribution of PageRank and CheiRank in Fig 2 between the “normal” and “cancer” regulatory network, we characterized the relative change of the ranks by computing their log ratio between two networks. Overall, the relative changes in CheiRank had larger amplitude than in PageRank which can be partially explained by the different number of transcriptional targets of the transcriptional factors, described in the GM12878 and K562 transcriptional networks.

**Fig 5:**
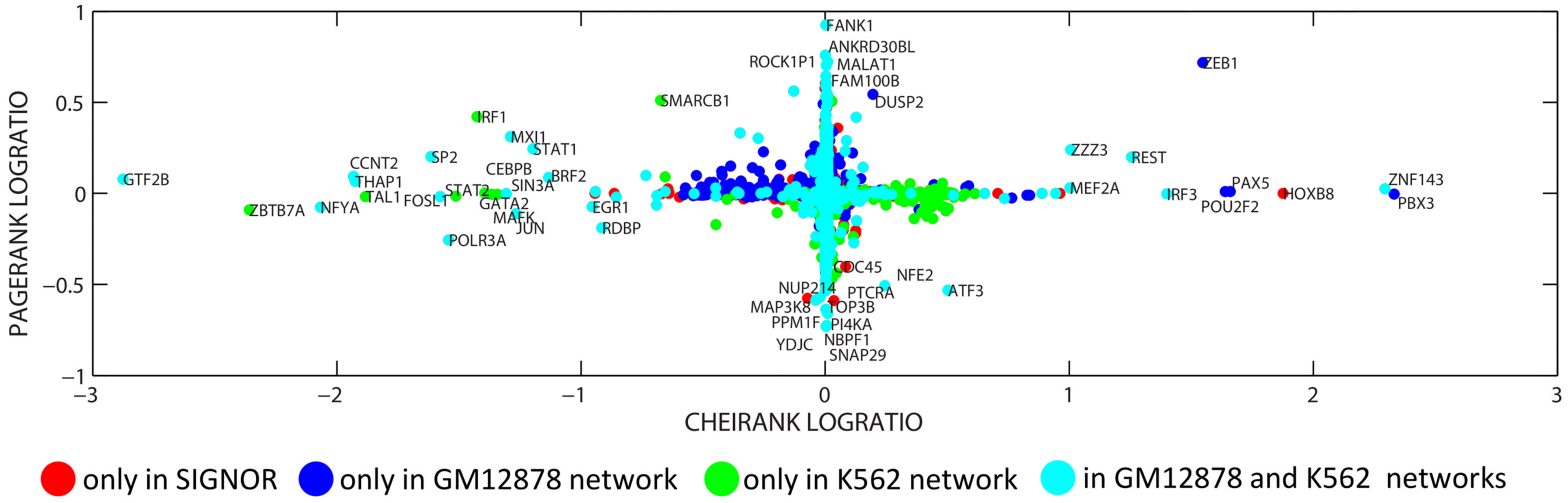
Relative changes in the PageRank and CheiRank in cancer vs normal network. The abscissa is the decimal logarithm of the ratio *K*_*K*562_/*K*_*GM*_, where the subscript denotes in which network the CheiRank is calculated. The ordinate is the decimal logarithm of the ratio $K_{K562}^{*}/K_{GM}^{*}$. For example, the proteins in the right part of the plot such as MEF2A, ZNF143, ZEB1 are those whose CheiRank significantly increased (i.e., the protein has less outgoing connections) in the “cancer network” K562 (which can be simply because the corresponding transcription factor is not present in K562 transcriptional network, as in the case of ZEB1, blue points in the plot).

We characterized the biological functions represented by those proteins significantly deviating from zero in Fig 5, by applying the standard enrichment analysis based on hypergeometric test, calculating what is the probability (p-value) of a selected protein set to intersect with some predefined reference protein set by random chance. For this purpose we used the *toppgene* bioinformatics package [45]. For the enrichment analysis, we took those proteins deviating from zero by two standard deviations of the distributions of PageRank of CheiRank log ratios, and analyzed the positive and negative sides of the distribution separately. The results of the analysis are presented in Table 1 and online at [46].

**Table 1:** Table of enriched biological functional categories of proteins. The numbers signifies the number of proteins whose ranks are significantly changed. By bold those functional categories are highlighted which found enriched after filtering out proteins not present in either cancer or normal transcriptional networks. In this case, two values for the number of proteins are shown separated by semicolon: one without filtering and one after filtering. Complete interactive version of this table is available at [46]. Arrow up means that the corresponding rank decreases (or, in other words, “improves”, rank value increases) in the cancer network (the proteins are more connected). Arrow down means the opposite: the corresponding rank “degrades” and the proteins become less connected in cancer.

Change of Rank in cancer	Number of proteins	Selected enriched functional categories/signatures
Page↑	158;86	GO:0004812: **aminoacyl-tRNA ligase activity (8;4)**
		GO:0006412: **translation (22;14)**
		GO:0005925: focal adhesion (11)
		Interactions: FBXO6 (22), ITGA4 (19), **CUL5(17;12)**
		Cytoband: **22q11.21 (9;7)**
		Transcription factor binding site: **V$E2F Q6 (9;6)**
		Disease: **Shprintzen syndrome (8;5)**
Page↓	158;63	GO:0090544: BAF-type complex (5)
		GO:0016514: SWI/SNF complex (4)
		Mouse phenotype: abnormal bone marrow cell morphology/development (17)
		Pathway: REACTOME Cell Cycle (16), TNF-alpha/NF-kB Signaling Pathway (9)
		Interactions: ARID2 (6), DPF3 (5)
		Transcription factor binding site: **TMTCGCGANR UNKNOWN (14;8)**
Chei↑	105;30	GO:0001047: **core promoter binding (15;8)**
		GO:0030097: **hemopoiesis (25;9)**
		GO:0034097: **response to cytokine (22;8)**
		GO:0045637: **regulation of myeloid cell differentiation (11;5)**
		GO:0000785: **chromatin (21;7)**
		Mouse phenotype: abnormal bone marrow cell morphology/development (25)
		Mouse phenotype: **increased lymphocyte cell number (23;9)**
		Pathway: KEGG Transcriptional misregulation in cancer (12)
		Pathway: WikiPathways EGFR1 Signaling Pathway (10)
		Pathway: *BIOCARTA MAPKinase Signaling Pathway* (;*5*)
		Interactions: **EP300 (28;14), TBP (13;10), JUN (19;11) SP1 (18;10), CREBBP (25;11), HDAC1 (28;12)**
		Co-expression: **Genes up-regulated in MCF7 cells (breast cancer) after stimulation with EGF (7;6)**
		Co-expression: **Genes regulated by NF-kB in response to TNF (15;7)**
		MicroRNA targets: **hsa-miR-548m:PITA (11;7)**
		Disease: **Myeloid Leukemia (20;9)**
Chei↓	83;23	GO:0000975: **regulatory region DNA binding (23;16)**
		GO:0048534: **hematopoietic or lymphoid organ development (17;9)**
		GO:0000785: **chromatin (14;9)**
		Mouse phenotype: **decreased thymocyte number (9;7)**
		Mouse phenotype: **increased apoptosis (19;10)**
		Pathway: **WikiPathways TGF-beta Receptor Signaling Pathway (8;6)**
		Interactions: **EP300 (23;10), RB1 (13;6)**
		Disease: **Adult T-Cell Lymphoma/Leukemia (13;7)**
		Disease: B-Cell Lymphomas (16)

We’ve noticed, however, that the results of this analysis can be biased by a simple fact that a protein can be included in the “normal” transcriptional network GM12878 (thus having, for example, many transcriptional out-going interactions) and not at all included in the “cancer” transcriptional network K562 (thus having no transcriptional out-going interactions at all). This is the case, for example, for ZEB1 transcriptional factor. Despite the fact that such difference can be “real”, i.e. the transcriptional factor might be not expressed in the case of cancer, hence, does not regulate any genes, we’ve decided to perform an additional analysis focusing only at those proteins which simultaneously present in both “normal” and “cancer” transcriptional networks. These proteins might be present or not in the SIGNOR network. This second analysis made the p-values for several biological functions detected in the previous analysis insignificant (normal text lines in the Table 1) but many remained significant (bold text lines in the Table 1). For the second analysis, in case of CheiRank, we took those proteins which deviated from zero by one standard deviation in the distribution of log ratio of the CheiRanks, in order to collect a sufficient number of proteins.

Overall, the undertaken analysis shows a picture consistent with the nature of the studied cells. I.e., we show that changes in the CheiRanks between normal and cancer cells highlights a number of proteins previously described as being implicated in leukemia (16 from 53 selected for the second analysis). Interestingly, this analysis highlights proteins implicated in the regulation of myeloid cell differentiation and hemopoiesis, which is also expected. 9 from 30 selected proteins were previously associated with a mouse phenotype characterized by the increased number of lymphocytes. They significantly improved their CheiRank in the cancer network (became more powerfull regulators). Having many transcriptional factors in the transcriptional networks explains significance of such Gene Ontologies as “core promoter binding” and “chromatin” in the analysis of CheiRank changes in both directions, and also interactions with key transcription co-regulators as EP300, HDAC1 and CREBBP.

At the same time, the analysis gives also some unexpected findings. For example, a number of proteins involved in translation (14 from 86 selected) or having the E2F transcription factor binding motif the promoter sequence (6 from 86) or being located in a specific genomic locus 22q11 (7 from 86) improved their PageRank in cancer network (meaning they became more regulated). 8 from 63 genes with a specific motif in their promoter sequences (TMTCGCGANR) showed significant increase in the PageRank (meaning they became less regulated in cancer) which was also an unexpected finding.

#### Comparing PageRank and CheiRank to other centrality measures

We compared PageRank and CheiRank measures of centrality to several other widely used centrality measures (in- and out-closeness, betweenness measures, network authorities and hubs from hyperlink-induced topic search), as it was discussed elsewhere [33–36]. Closeness centrality is defined as the sum of the length of the shortest paths between the node and all other nodes in the graph. Obviously, it can distinguish incoming and outgoing paths. Betweenness centrality is defined as the number of shortest paths between all nodes in the network passing through a given node. Unlike closeness, betweenness can not distinguish incoming and outgoing paths.

Firstly, we’ve computed the Spearman correlations for each pair of comparable centrality measures (see S1 Fig). From this analysis it was clear that PageRank is very weakly correlated to other centrality measures (besides simple indegree), in the case of a directed graph representing the SIGNOR signaling network. Quite opposite, the correlations were much stronger in the case of CheiRank compared to out-closeness and hub measures. Betweenness was weakly correlated to both PageRank and CheiRank which can be explained by its “non-oriented” nature.

Secondly, we performed the same analysis as reported in the previous section, by applying the term enrichment analysis to the changes in the closeness, betweenness and authority/hub measures, in normal and cancer networks. This analysis showed that betweenness change was able to identify several of the meaningful terms listed in Table 1; for example, groups of genes involved in Myeloid Leukemia (p-value 10^−7^). However, it did not distinguish between the nature of the changes (excess/deficit of incoming or outgoing regulations in cancer) and was not able to identify groups of genes related to cell cycle (which was significant in the PageRank analysis). By contrast, in-closeness was able to capture the disregulation of the cell cycle (p-value 10^−5^ for G2/M Transition term from REACTOME pathway database) while neither in- or out-closeness identified the nature of the disease (leukemia). Both authority and hub centrality measures were not informative in their changes in cancer compared to normal cells and collected very few and unrelated enriched terms.

The two above-mentioned analyses allow us to conclude that PageRank and CheiRank represent informative measures of network centrality which can be applied to identify structural changes in the oriented biological networks in diseases such as cancer. Both PageRank and CheiRank can be more informative than other centrality measures for interpretation of these changes. PageRank contains more complementary information compared to other centrality measures than CheiRank.

### Inferring hidden causal relations between members of a protein set

Application of Google matrix to the global network allows quantifying the global ranking of protein nodes and their changes, as it was illustrated in the previous section. Reduced Google matrix (see Materials and Methods for formal description) allows focusing on a subset of nodes, quantify local importance of nodes in this subset and also detect indirect (hidden) connections between the members of the subset.

In order to test this approach in the context of biological networks, we’ve defined several protein sets, each of which contains a functionally related group of proteins. However, the meaning of the functional proximity was different in all cases. When defining these sets, we used only general considerations, and not any informed decision based on preliminary analysis.

We start with a definition of a biological pathway, which play one of the most central roles in all cancer types: AKT-mTOR pathway. It is a molecular cascade downstream of PI3K kinase which is important in regulating the cell cycle and cell survival, controlling a number of normal physiological processes, and being dysregulated in many diseases including cancer. We take the definition of the AKT-mTOR pathway from the external pathway database Atlas of Cancer Signaling Network (ACSN) [47], based on manual mining for interactions from thousands of molecular biology publications, so the definition of this subset of proteins can be called “knowledge-driven”.

Second analyzed subset by contrast was chosen to be purely “data-driven” and corresponds to a particular gene expression signature (set of genes), shown to be connected to cell proliferation in multiple cancer studies through data analysis. We used a particular definition of this signature coming from a large multi-cancer study of tumoral transcriptomes, using Independent Component Analysis (ICA) method [48, 49]. Gene expression signatures obtained through statistical data analysis sometimes serve as a scaffold for reconstructing the topology of regulatory connections between the corresponding proteins.

Third analyzed group of proteins was chosen to be a set of known direct targets of a transcriptional factor E2F1 which is central to regulation and progression through the cell cycle. The member names of this set were manually extracted from reading the molecular biology literature on the functioning of cell cycle in order to reconstruct it as a biochemical reaction diagram [50]. In this case, the challenge is to understand what biological pathways can be directly regulated by E2F1, what are the possible direct and indirect feedbacks to the regulation of E2F1 itself and how they are changing in cancer progression.

All three subsets are central to the studied in this paper cancer progression and its influence on the structure of biological networks. In all three cases, we roughly equilibrated the sizes of the protein sets, limiting them to approximately 50 proteins.

It happened that the direct interactions between the members of all three sets of proteins are described in SIGNOR pathway database, and not in the transcriptional networks. Therefore, one might consider that the wiring of direct connections between the set members are not affected by the changes in the transcriptional program. However, we further show that the structure of indirect connections might change accordingly to the changes in the global context created by the transcriptional network.

#### AKT-MTOR pathway

From SIGNOR database, we’ve retrieved 138 direct regulatory connections between 63 proteins of AKT-mTOR pathway. These direct connections formed a large connected subnetwork containing the majority (43 proteins) of the pathway members, and the rest was orphan nodes not connected to any other.

No direct transcriptional regulatory connections was found between the members of the pathway; hence, the structure of direct connections did not change in the “cancer” network with respect to the “normal” network.

We’ve computed indirect regulatory relations using the reduced Google matrix approach as described in Materials and Methods section, separately for SIGNOR, the “normal” and “cancer” global regulatory networks, combining the common signaling SIGNOR part and the specific transcriptional network. The strength of the indirect regulation can be evaluated by looking at its *G*_qr_ value.

**Fig 6:**
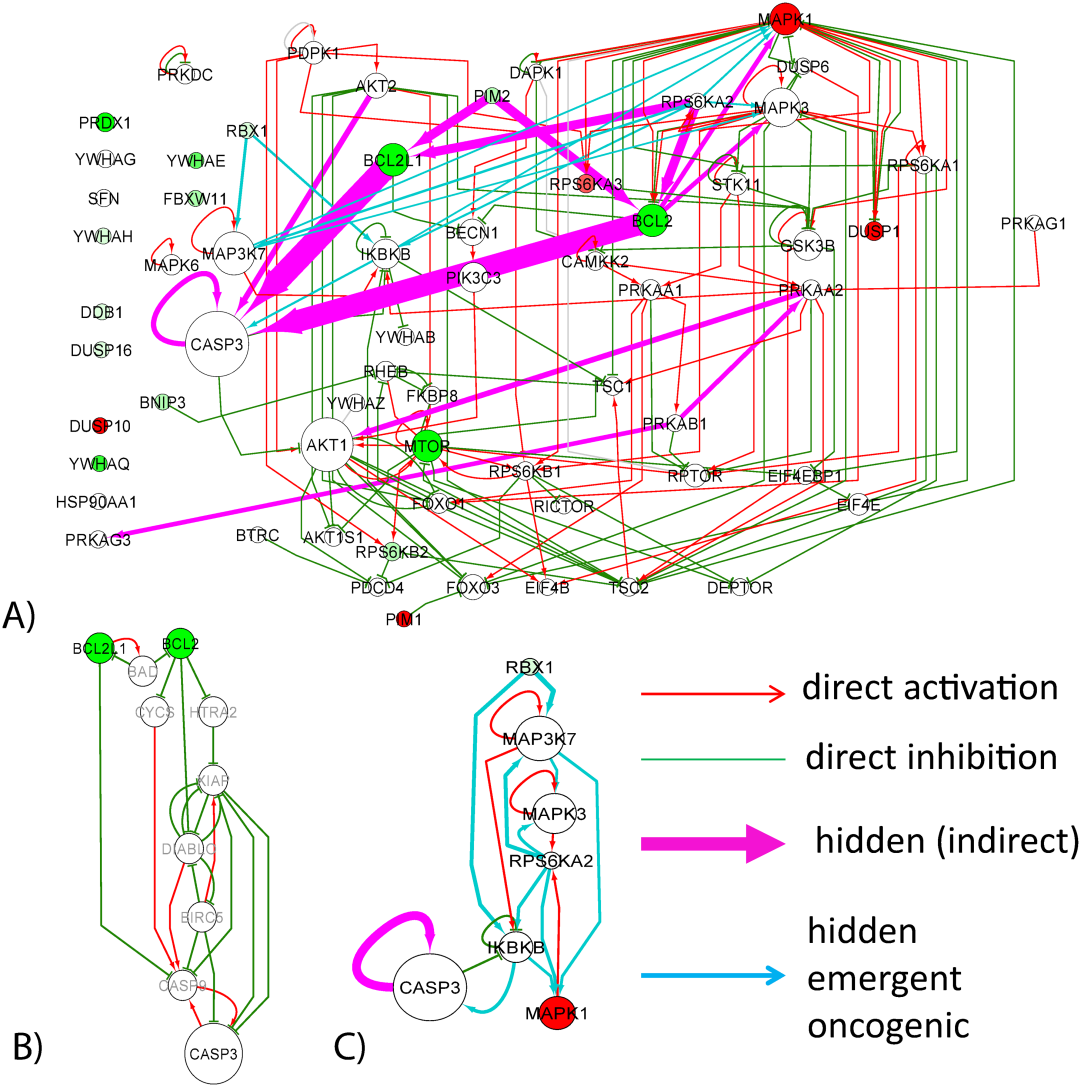
AKT-mTOR pathway reconstructed using SIGNOR database and by inferring indirect connections using reduced Google matrix approach. A) Direct connections representing activator and inhibitor regulations are shown as red and green arrows correspondingly. Magenta color arrows shows the inferred indirect interactions which are common between the “normal” and “cancer” networks. Light blue color arrows represent those indirect interactions which are inferred only in the “cancer” network. Line width of the arrows representing the indirect interactions, is proportional to their *G*_qr_ score. Here only 10 top scored indirect connections present in both “normal” and “cancer” networks and 10 top “emergent in cancer” connections are shown. The color of protein nodes reflects the relative change in their local PageRanks in “cancer” vs “normal” networks. Red color means that the protein become better ranked in PageRank in the “cancer” network, and the green color means the opposite. The size of the node is proportional to the value of the PageRank in the SIGNOR network. B) Hidden cascade of indirect regulations connecting BCL2 and BCL2L1 proteins with CASP3. The proteins shown with grey labels are those which are not present in the definition of AKT-mTOR pathway. C) Cascade of hidden interactions emerging in the “cancer” network and not present in “normal” network.

For the “normal” network we found that the distribution of the corresponding $G_{\text{qr}}^{\left(GM\right)}$ values contains essentially close to zero values, with only some pointing to existence of indirect regulation. Thus, for an arbitrarily chosen threshold *G*_qr_ > 0.01 one detects 50 indirect interactions, ten top of them are shown in Fig 6A (in magenta color). These 50 indirect regulations connected 8 more proteins to the largest connected component.

It can be noticed that the pattern of the top indirect connections is highly non-random and forms two “hidden pathways”, one pointing to CASP3 protein through BCL proteins, and one connecting PRKA proteins to AKT1. Both hidden pathways have rather clear biological interpretation.

The first one can be related to the existence of apoptotic pathway in the global regulatory network, where BCL2 and BCL2L1 proteins play an important role. CASP3 serves the final point of the apoptotic pathway, being the main executor protein of the apoptotic process (the executor caspases take care of destroying the proteins of the suicided cell and the cell itself). In order to illustrate how BCL proteins and CASP3 are connected through the global network, we’ve computed the shortest and the second shortest oriented path between BLC2 and BCL2L1 proteins and CASP3 protein (Fig 6B). It can be seen that these paths include the main players of the apoptotic machinery (XIAP, CASP9, DIABLO, CYCS). Second hidden pathway connects the subunits of AMP-activated protein kinase (AMPK), an important energy sensor protein, to AKT1.

The hidden regulations also point out to the important crosstalk between BCL2 and MAPK1, MAPK3 proteins, not represented by direct interactions inside AKT-mTOR pathway.

As a conclusion, one can state that the reduced Google matrix approach was able to point out to biologically important and meaningful indirect connections between several AKT-mTOR pathway members.

In addition, we compared the inferred indirect interactions in “normal” and “cancer” global networks. We found that there is a strong correlation between all three set of values $G_{\text{qr}}^{\left(GM\right)},G_{\text{qr}}^{\left(K562\right)},G_{\text{qr}}^{\left(SIGNOR\right)}$ (correlation coefficients are close to 0.998). All strong indirect interactions inferred using the “normal” GM network were also found in “cancer” K562 network. However, in the “cancer” network we found additional candidates for indirect interactions, top ten of which are shown in Fig 6A and 6C. It can be seen that such “emergent oncogenic” indirect interactions also underline existence of a “hidden” causal relation between RBX1 and MAPK1 proteins.

#### Data-driven signature of proliferation-related proteins

We analyzed a set of 49 proteins found in SIGNOR database whose expression was shown to significantly change between fast proliferative and slow proliferative tumors in 9 cancer types [49]. We found 47 direct interactions connecting them into one large connected component consisting of 31 proteins who were predominantly the phosphorylation targets of the cyclin-dependent kinase CDK1, so the structure of the network of direct interactions is fan-like organized around one large hub protein. As in the previous example, no direct transcriptional connections were found between the members of this protein set.

**Fig 7:**
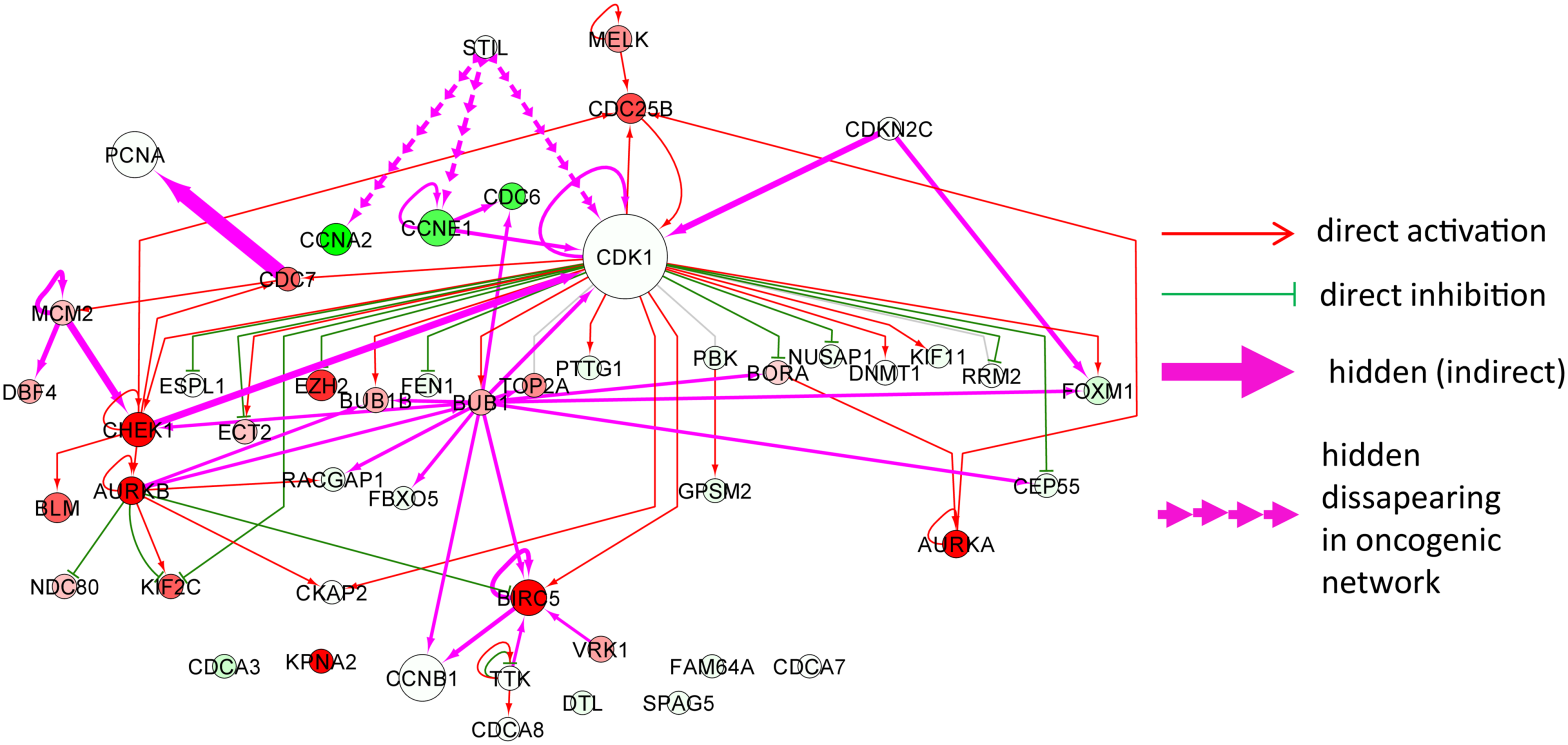
Network of proteins shown to be related to cell proliferation by transcriptomic data analysis. The meaning of the node and edge colors is the same as in Fig 6 besides those regulations which disappear in “cancer” network compared to “normal” network (shown by interrupted line arrows).

We’ve computed indirect regulatory relations using the reduced Google matrix approach as described in Materials and Methods section, separately for SIGNOR, the “normal” and “cancer” global regulatory networks, combining the common signaling SIGNOR part and the specific transcriptional network. As before, we’ve found only a minor fraction of all pair-wise protein relations as candidates for indirect interactions (only 32 from 2305 passed the threshold *G*_qr_ > 0.01). In Fig 7 we show all indirect regulations inferred in the “normal” GM network. As before, with indirect connections, it was possible to connect more proteins (43 out of 49). For example, PCNA protein was connected to the largest connected component of the network. PCNA (proliferating cell nuclear antigen) protein is a key cell cycle protein important both for DNA replication and DNA repair.

While comparing “cancer” and “normal” networks, unlike the previous example, we do not find new “emergent oncogenic” indirect interactions. Instead, we observed that several indirect interactions disappear in the “cancer” network, namely three indirect regulations connecting STIL protein to CCNA2, CCNE1 and CDK1. STIL is a cytoplasmic protein implicated in regulation of the mitotic spindle checkpoint, a regulatory pathway that monitors chromosome segregation during cell division to ensure the proper distribution of chromosomes in daughter cells. Interestingly, STIL protein was shown to be heavily deregulated in T-cell leukemias through genome modifications leading to gene fusions. Disappearance of indirect connections between STIL and CDK1 can be interpreted as loosening the control over several important cyclins (CCNA2 and CCNE1) and the key cell cycle protein CDK1 in cancer. Consistently, we find that the PageRank of the aforementioned cyclins increases (e.g., for the local subnetwork PageRanks, $K_{CCNA2}^{GM}=7$ and $K_{CCNA2}^{K562}=14$) which means that they are less regulated/controlled in cancer. Several other proteins such as AURKA, AURKB, CHEK1, BIRC5, CDC25B decreases their local PageRanks in cancer which means they become more controlled. Interestingly, one of the direct targets of CDK1 kinase, protein BUB1, becomes a new hub of the indirect interactions. Indeed, this protein plays a central role in mitosis by phosphorylating members of the mitotic checkpoint complex and activating the spindle checkpoint.

#### Set of transcriptional targets of E2F1 transcription factor

In our last example, we used the reduced Google matrix method in order to better understand the structure of regulations of known in advance direct targets of a selected transcription factor E2F1, a key transcription factor regulating cell cycle progression. For 76 such proteins found in SIGNOR pathway database, we find 103 direct interaction connecting these proteins into the largest connected component comprising 49 proteins. One additional direct transcriptional regulation was found between MYC and CBX5 proteins, but only in “cancer” K562 network.

**Fig 8:**
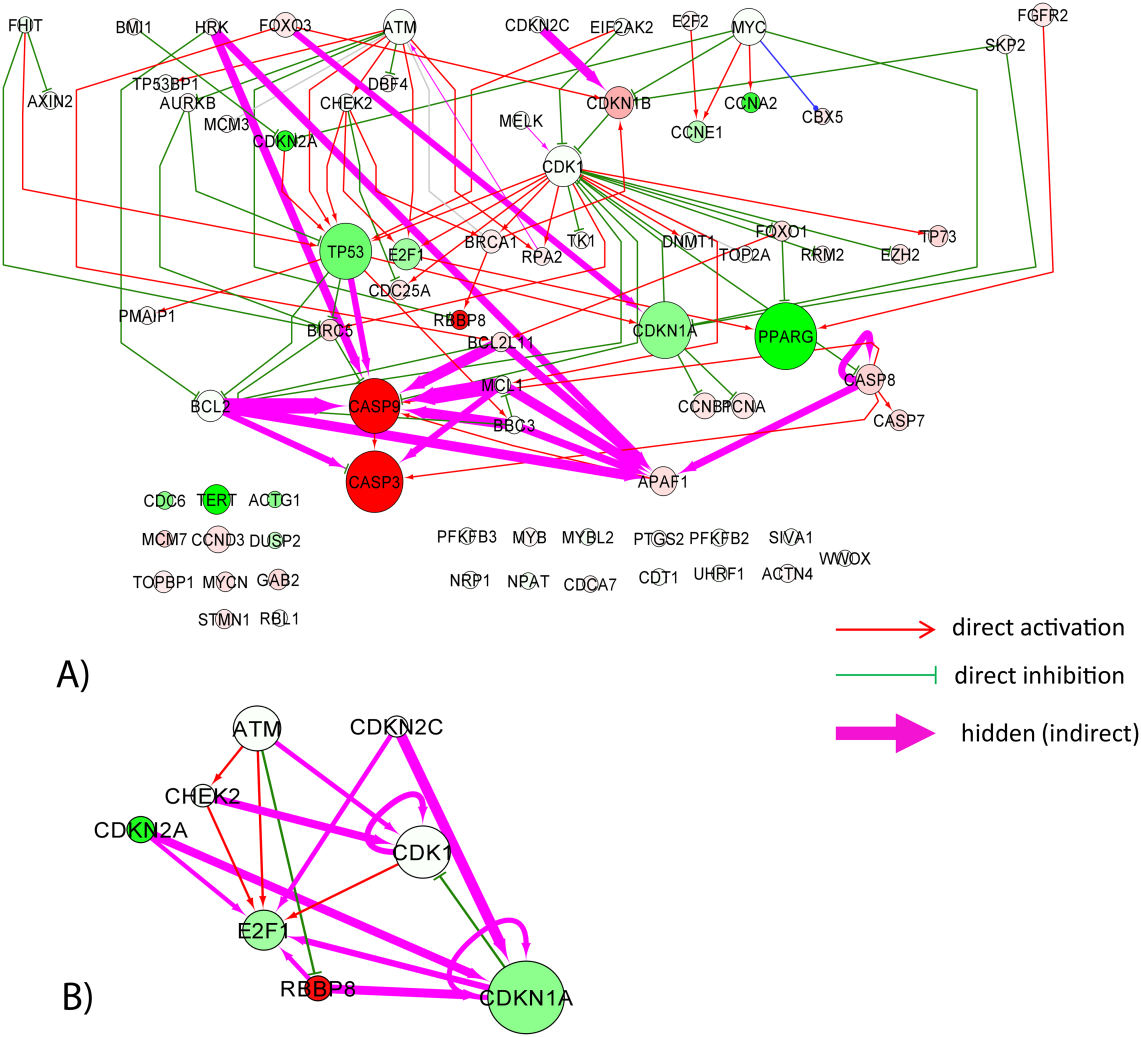
Network of targets of E2F1 transcription targets. The meaning of the node and edge colors is the same as in Fig 6. A) Complete network with a subset of 19 top scored indirect interactions is shown for *G*_qr_ > 0.05. B) Network of E2F1 direct and indirect (*G*_qr_ > 0.01) regulators of E2F1, showing multiple indirect feedback loops in the regulation of E2F1 itself.

The reduced Google matrix analysis revealed 84 indirect regulations for *G*_qr_ > 0.01 in the case of the “normal” network (Fig 8A), all of which were also present in the “cancer” network. We did not find any additional indirect interactions from the analysis of the “cancer” network.

The majority of strong indirect interactions pointed to the 3 key apoptosis proteins CASP9, CASP3 and APAF1 (apoptotic protease activating factor) whose local PageRanks decreased in the “cancer” network (which means they become more regulated). As in the AKT-mTOR example, we find indirect regulations between BCL2, BCL2L1 and CASP3. However, unlike AKT-mTOR example, we did not observe significant changes of local PageRanks of BCL2 and BCL2L1. Overall, the reduced Google matrix analysis underlines existence of hidden indirect apoptotic program regulated by E2F1 (which is a known fact [51]).

We also found that many weaker indirect interactions between the targets of E2F1 ends up on the E2F1 itself (Fig 8B), also through a key G1/S cell cycle checkpoint protein CDKN1A (cyclin dependent kinase inhibitor 1A). Therefore, E2F1 itself can be regulated through a number of direct (3 in Fig 8B) and even more indirect (4 in Fig 8B) feedback regulations. This observation can provide hints on the principles of organization of the cell cycle transcriptional program.

## Materials and methods

### Google matrix construction and properties

The Google matrix *G* of a directed network of *N* nodes is constructed from the adjacency matrix *A*_*ij*_ which has elements 1 if a protein (node) *j* points to a protein (node) *i* and zero otherwise. Then the matrix elements of *G* take the standard form [13, 33]

$$\begin{array}{c} G_{ij}=\alpha S_{ij}+\left(1-\alpha \right)/N\;\;, \end{array}$$ (1)

where *S* is the matrix of Markov transitions with elements *S*_*ij*_ = *A*_*ij*_/*k*_*out*_(*j*), $k_{out}\left(j\right)=\sum_{i=1}^{N}A_{ij}\neq 0$ being the node *j* out-degree (number of outgoing links) and with *S*_*ij*_ = 1/*N* if *j* has no outgoing links (dangling node). Here 0 < *α* < 1 is the damping factor which for a random surfer determines the probability (1 − *α*) to jump to any node. The properties of spectrum and eigenstates of *G* have been discussed in detail for Wikipedia and other directed networks (see e.g. [18]).

The right eigenvectors *ψ*_*i*_(*j*) of *G* are determined by the equation:

$$\begin{array}{c} \sum_{j^{\prime }}G_{jj^{\prime }}\psi_{i}\left(j^{\prime }\right)=\lambda_{i}\psi_{i}\left(j\right)\;. \end{array}$$ (2)

The PageRank eigenvector *P*(*j*) = *ψ*_*i* = 0_(*j*) corresponds to the largest eigenvalue λ_*i* = 0_ = 1 [13, 33]. It has positive elements which give a probability to find a random surfer on a given node in the stationary long time limit of the Markov process. All nodes can be ordered by a monotonically decreasing probability *P*(*K*_*i*_) with the highest probability at *K* = 1. The index *K* is the PageRank index. Left eigenvectors are biorthogonal to right eigenvectors of different eigenvalues. The left eigenvector for λ = 1 has identical (unit) entries due to the column sum normalization of *G*. One can show that the damping factor *α* in (1) only affects the PageRank vector (or other eigenvectors for λ = 1 of *S* in case of a degeneracy) while other eigenvectors are independent of *α* due to their orthogonality to the left unit eigenvector for λ = 1 [33]. Thus all eigenvalues, except λ = 1, are multiplied by a factor *α* when replacing *S* by *G*. In the following we use the notations $\psi_{L}^{T}$ and *ψ*_*R*_ for left and right eigenvectors respectively (here *T* means vector or matrix transposition).

In many real networks the number of nonzero elements in a column of *S* is significantly smaller than the whole matrix size *N* that allows to find efficiently the PageRank vector by the PageRank algorithm of power iterations [33]. Also a certain number of largest eigenvalues (in modulus) and related eigenvectors can be efficiently computed by the Arnoldi algorithm (see [18] and Refs. therein).

In addition to the matrix *G* it is useful to introduce a Google matrix G* constructed from the adjacency matrix of the same network but with inverted direction of all links. The statistical properties of the eigenvector P* of G* with the largest eigenvalue λ = 1 have been studied first for the Linux Kernel network [52] showing that there are nontrivial correlations between *P* and P* vectors of the network. More detailed studied have been done for Wikipedia and other networks [18]. The vector P*(K*) is called the CheiRank vector and the index numbering nodes in order of monotonic decrease of probability P* is noted as CheiRank index K*. Thus, nodes with many ingoing links have small value of *K* = 1, 2, 3… and nodes with many outgoing links have K* = 1, 2, 3, … [18, 33]. Examples of density distributions for Wikipedia editions and other directed networks are given in [18]. It is also useful to use 2DRank index *K*_2_ which represents a certain combination of *K*, K* indexes (*K*_2_ is the sequence of *K*, K* values appearing first on a sequence of squares which have left corner at *K* = K* = 1 with size increasing one by one up to maximal *N* value, see details in [18]). Similar to the PageRank vector the CheiRank vector gives probabilities to find a random surfer on a given node (for a network with inverted direction of links) in the limit of very large number of jumps. On average these probabilities are proportional to the number of outgoing links.

At *α* < 1 only the PageRank vector have λ = 1 while all other eigenvectors of *G* have |λ| ≤ *α* [18, 33]. For Wikipedia is was shown that the eigenvectors with a large modulus of λ select some specific communities of Wikipedia network [18]. However, a priory it is not possible to know what are the meanings of these communities. Thus other methods are required to determine effective interactions between *N*_*r*_ nodes of a specific subset (group) of the global network of a large size *N* ≫ *N*_*r*_.

In this work we apply the Google matrix analysis to the directed network of protein interactions from the cancer database SIGNOR [7], and two hybrid networks, constructed by merging SIGNOR to two transcriptional networks measured in normal blood cells and in cancer (leukemia). The SIGNOR directed network contains *N* = 2432 proteins (nodes) with the total number of links *N*_*ℓ*_ = 6569. In all our analysis we use the typical damping factor value *α* = 0.85 [33].

**Fig 9:**
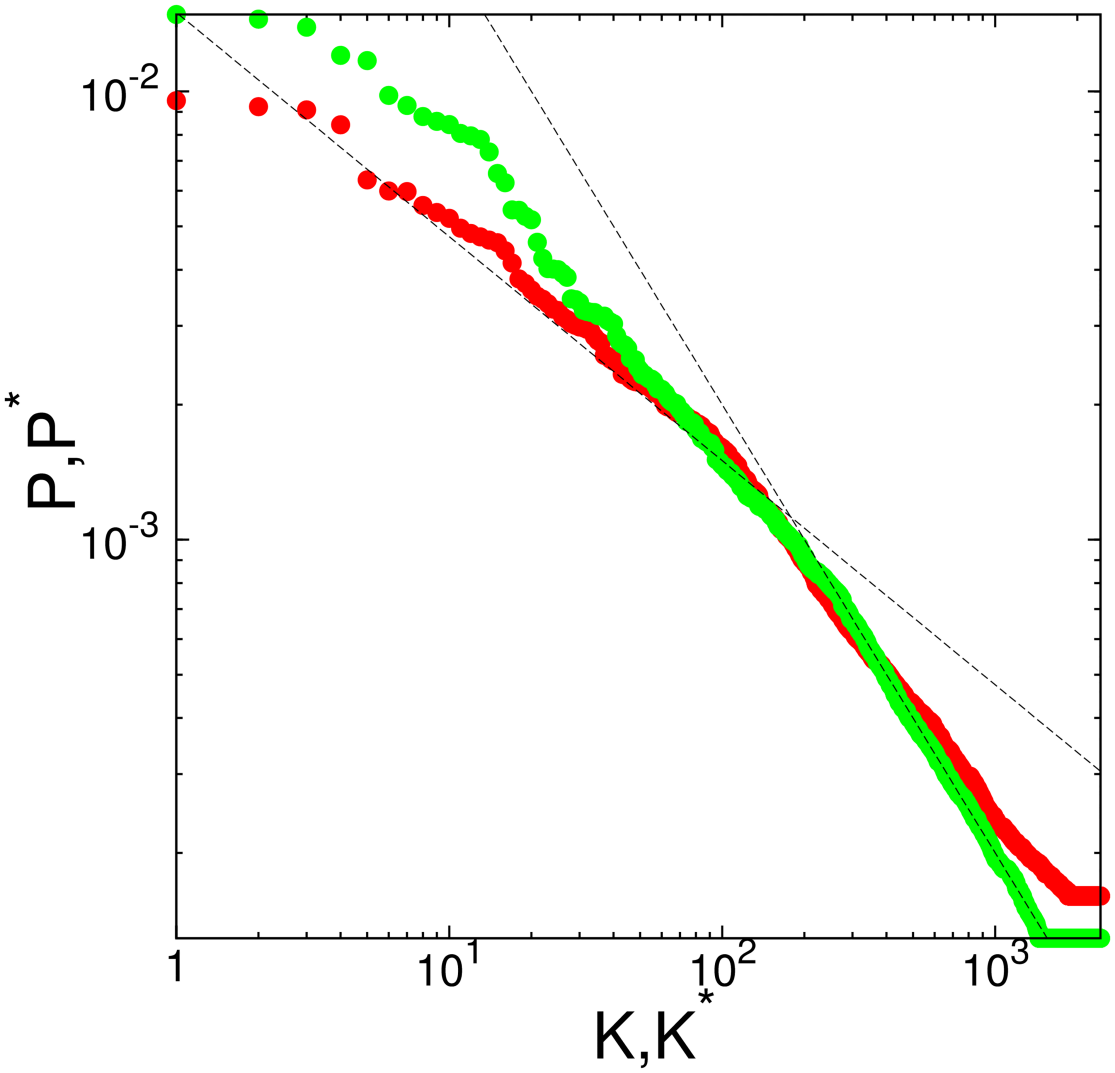
PageRank probability *P* (red points) and CheiRank probability P* (green points) as a function of *K* and K* indexes, calculated for SIGNOR molecular interaction database. Straight dashed lines are drawn to adapt an eye and show algebraic decay with exponents −0.5 and −1. Here *α* = 0.85.

**Fig 10:**
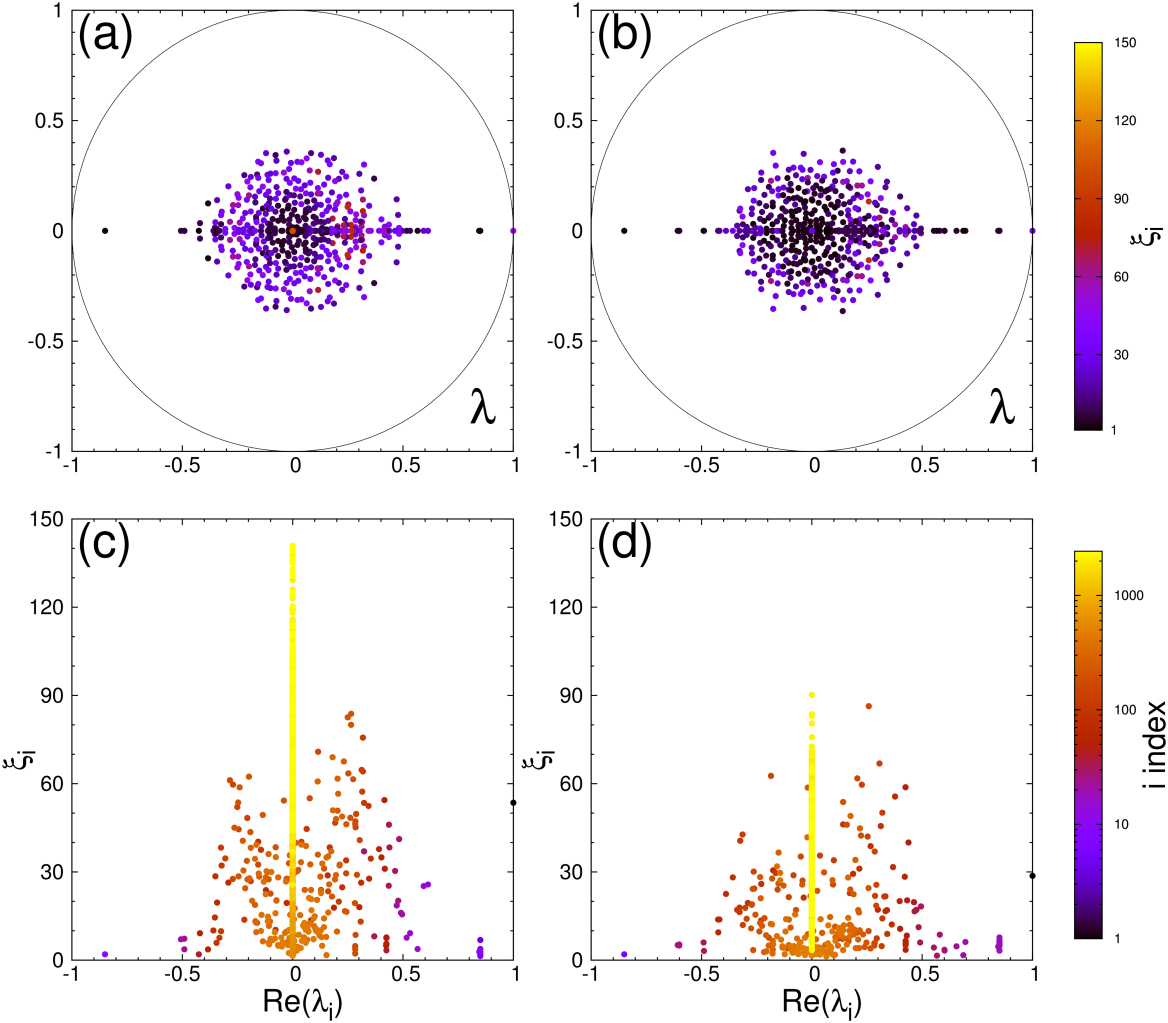
Spectral analysis of Google matrix for SIGNOR database. (a, b) Complex spectrum {λ_*i*_}_*i* = 0,…,*N*−1_ of Google matrices *G* (a) and G* (b) at *α* = 0.85, computed for SIGNOR molecular interaction database. Colors give the inverse participation ratio *ξ*_*i*_ from *ξ*_*i*_ = 1 (black) to *ξ*_*i*_ = 150 (bright yellow). (c, d) Inverse participation ratio $\xi_{i}=(\sum_{n=1}^{N}{\left|\psi_{i}\left(n\right)\right|}^{2})/\sum_{n=1}^{N}{\left|\psi_{i}\left(n\right)\right|}^{4}$ of the *i*th eigenvector as a function of *Re* (λ_*i*_) for Google matrices *G* (c) and G* (d). Colors give in logarithmic scale *i* indexes of eigenvalues ranked by decreasing modulus; from *i* = 1 (black) to *i* = *N* = 2432 (bright yellow). Quasi-uniform picture of the spectrum reflects inexistence of weak protein node communities in the SIGNOR network.

For the studied protein networks the dependencies of PageRank and CheiRank probabilities on rank indexes are shown in Fig 9. The decay of probabilities is approximately described by a power law *P* ∝ 1/*K*^*β*^; P* ∝ 1/K*^β^ with the decay exponent *β* in a range 0.5 − 1. However, this is only an approximation for a whole curve. The distribution on nodes on the PageRank-CheiRank plane is shown in Fig 2. The spectra of *G* and G* are shown in Fig 10.

### Reduced Google matrix

Recently, the method of reduced Google matrix has been proposed for analysis of effective interactions between nodes of a selected subset embedded into a large size network [37]. This approach uses parallels with the quantum scattering theory, developed for processes in nuclear and mesoscopic physics and quantum chaos.

It turns out that the Google matrix *G*_R_ matrix describing the interactions inside a group of proteins is composed of three matrix components which describe the direct interactions between group members, *G*_*rr*_, a projector part *G*_*pr*_ which is mainly imposed by the PageRank of selected proteins given by the global *G* matrix and a component *G*_qr_ from hidden interactions between proteins which appear due to indirect links via the global network. Thus the reduced matrix *G*_R_ = *G*_*rr*_ + *G*_pr_ + *G*_qr_ allows to obtain precise information about the group of proteins taking into account their environment given by the global network.

The concept of reduced Google matrix *G*_*R*_ was introduced in [37] on the basis of the following observation. At present directed networks of real systems can be very large (about 4.2 millions for the English Wikipedia edition in 2013 [18] or 3.5 billion web pages for a publicly accessible web crawl that was gathered by the Common Crawl Foundation in 2012 [53]). In certain cases one may be interested in the particular interactions among a small reduced subset of *N*_*r*_ nodes with *N*_*r*_ ≪ *N* instead of the interactions in the entire network. However, the interactions between these *N*_*r*_ nodes should be correctly determined taking into account that there are many indirect links between the *N*_*r*_ nodes via all other *N*_*s*_ = *N* − *N*_*r*_ nodes of the network. This leads to the problem of the reduced Google matrix *G*_*R*_ with *N*_*r*_ nodes which describes the interactions of a subset of *N*_*r*_ nodes.

In a certain sense we can trace parallels with the problem of quantum scattering appearing in nuclear and mesoscopic physics [54–56] and quantum chaotic scattering [57]. Indeed, in the scattering problem there are effective interactions between open channels to localized basis states in a well confined scattering domain where a particle can spend a certain time before its escape to open channels. Having this analogy in mind we construct the reduced Google matrix *G*_*R*_ which describes interactions between selected *N*_*r*_ nodes and satisfies the standard requirements of the Google matrix.

Let *G* be a typical Google matrix of Perron-Frobenius type for a network with *N* nodes such that *G*_*ij*_ ≥ 0 and the column sum normalization $\sum_{i=1}^{N}G_{ij}=1$ are verified. We consider a sub-network with *N*_*r*_ < *N* nodes, called “reduced network”. In this case we can write *G* in a block form:

$$\begin{array}{c} G=(\begin{array}{cc} G_{rr} & G_{rs}\\ G_{sr} & G_{ss} \end{array}) \end{array}$$ (3)

where the index “*r*” refers to the nodes of the reduced network and “*s*” to the other *N*_*s*_ = *N* − *N*_*r*_ nodes which form a complementary network which we will call “scattering network”.

We denote the PageRank vector of the full network as

$$\begin{array}{c} P=(\begin{array}{c} P_{r}\\ P_{s} \end{array}) \end{array}$$ (4)

which satisfies the equation *GP* = *P* or in other words *P* is the right eigenvector of *G* for the unit eigenvalue. This eigenvalue equation reads in block notations:

$$\begin{array}{ccc} \left(1-G_{rr}\right)\;P_{r}-G_{rs}\;P_{s} & = & 0, \end{array}$$ (5)

$$\begin{array}{ccc} -G_{sr}\;P_{r}+\left(1-G_{ss}\right)\;P_{s} & = & 0. \end{array}$$ (6)

Here **1** is a unit diagonal matrix of corresponding size *N*_*r*_ or *N*_*s*_. Assuming that the matrix **1** − *G*_*ss*_ is not singular, i.e. all eigenvalues *G*_*ss*_ are strictly smaller than unity (in modulus), we obtain from (6) that

$$\begin{array}{c} P_{s}={\left(1-G_{ss}\right)}^{-1}G_{sr}\;P_{r} \end{array}$$ (7)

which gives together with (5):

$$\begin{array}{c} G_{R}P_{r}=P_{r}\;,\;G_{R}=G_{rr}+G_{rs}{\left(1-G_{ss}\right)}^{-1}G_{sr} \end{array}$$ (8)

where the matrix *G*_R_ of size *N*_*r*_ × *N*_*r*_, defined for the reduced network, can be viewed as an effective reduced Google matrix. Here the contribution of *G*_*rr*_ accounts for direct links in the reduced network and the second term with the matrix inverse corresponds to all contributions of indirect links of arbitrary order. We note that in mesoscopic scattering problems one typically uses an expression of the scattering matrix which has a similar structure where the scattering channels correspond to the reduced network and the states inside the scattering domain to the scattering network [55].

The matrix elements of *G*_*R*_ are non-negative since the matrix inverse in (8) can be expanded as:

$$\begin{array}{c} {\left(1-G_{ss}\right)}^{-1}=\sum_{l=0}^{\infty }G_{ss}^{\;l}\;\;. \end{array}$$ (9)

In (9) the integer *l* represents the order of indirect links, i. e. the number of indirect links which are used to connect indirectly two nodes of the reduced network. The matrix inverse corresponds to an exact resummation of all orders of indirect links. According to (9) the matrix (**1** − *G*_*ss*_)^−1^ and therefore also *G*_R_ have non-negative matrix elements. It can be shown that *G*_R_ also fulfills the condition of column sum normalization being unity [37].

The results obtained in [37, 38] show that the reduced Google matrix can be presented as a sum of three components

$$\begin{array}{c} G_{R}=G_{rr}+G_{\text{pr}}+G_{\text{qr}}, \end{array}$$ (10)

with the first component *G*_*rr*_ given by direct matrix elements of *G* among the selected *N*_*r*_ nodes, the second projector component *G*_pr_ is given by

$$\begin{array}{c} G_{\text{pr}}=G_{rs}P_{c}G_{sr}/\left(1-\lambda_{c}\right),\;P_{c}=\psi_{R}\psi_{L}^{T}\;. \end{array}$$ (11)

Here λ_*c*_ is the leading eigenvalue and by *ψ*_*R*_ ($\psi_{L}^{T}$) the corresponding right (left) eigenvector such that *G*_*ss*_ *ψ*_*R*_ = λ_*c*_ *ψ*_*R*_ (or $\psi_{L}^{T}G_{ss}=\lambda_{c}\psi_{L}^{T}$). Both left and right eigenvectors as well as λ_*c*_ can be efficiently computed by the power iteration method in a similar way as the standard PageRank method. We note that one can easily show that λ_*c*_ must be real and that both left/right eigenvectors can be chosen with positive elements. Concerning the normalization for *ψ*_*R*_ we choose $E_{s}^{T}\psi_{R}=1$ and for *ψ*_*L*_ we choose $\psi_{L}^{T}\psi_{R}=1$ (the vector $E_{s}^{T}$ has all elements being unity). It is well known (and easy to show) that $\psi_{L}^{T}$ is orthogonal to all other right eigenvectors (and *ψ*_*R*_ is orthogonal to all other left eigenvectors) of *G*_*ss*_ with eigenvalues different from λ_*c*_. Here we introduce the operator $P_{c}=\psi_{R}\psi_{L}^{T}$ which is the projector onto the eigenspace of λ_*c*_ and we denote by $Q_{c}=1-P_{c}$ the complementary projector. One verifies directly that both projectors commute with the matrix *G*_*ss*_ and in particular $P_{c}G_{ss}=G_{ss}P_{c}=\lambda_{c}P_{c}$.

We mention that this contribution is of the form $G_{\text{pr}}={\tilde{\psi }}_{R}{\tilde{\psi }}_{L}^{T}/\left(1-\lambda_{c}\right)$ with ${\tilde{\psi }}_{R}=G_{rs}\;\psi_{R}$ and ${\tilde{\psi }}_{L}^{T}=\psi_{L}^{T}G_{sr}$ being two small vectors defined on the reduced space of dimension *N*_*r*_. Therefore *G*_pr_ is indeed a (small) matrix of rank one which is also confirmed by a numerical diagonalization of this matrix. The third component *G*_qr_ of indirect or hidden links is given by

$$\begin{array}{c} G_{\text{qr}}=G_{rs}\left[Q_{c}\sum_{l=0}^{\infty }{\bar{G}}_{ss}^{\;l}\right]G_{sr},\;Q_{c}=1-P_{c},\;{\bar{G}}_{ss}=Q_{c}G_{ss}Q_{c}. \end{array}$$ (12)

Even though the decomposition (10) is at first motivated by the numerical efficiency to evaluate the matrix inverse it is equally important concerning the interpretation of the different terms and especially the last contribution (12) which is typically rather small as compared to (11) plays in an important role as we will see below.

Concerning the numerical algorithm to evaluate all contributions in (10), we mention that we first determine by the power iteration method the leading left *ψ*_*L*_ and right eigenvector *ψ*_*R*_ of the matrix *G*_*ss*_ which also provides an accurate value of the corresponding eigenvalue λ_*c*_ or better of 1 − λ_*c*_ (by taking the norm of the projection of *Gψ*_*R*_ on the reduced space which is highly accurate even for λ_*c*_ close to 1). These two vectors provide directly *G*_pr_ by (11) and allow to numerically apply the projector $Q_{c}$ to an arbitrary vector (with ∼*N* operations). The most expensive part is the evaluation of the last contribution according to (12). For this we apply successively *G*_*ss*_ and $Q_{c}$ to an arbitrary column of *G*_*sr*_ which can be done by a sparse matrix vector multiplication or the efficient application of the projector. We compute simultaneously the series in (12) which converges rather quickly after about 200 terms since the contribution of the leading eigenvalue (of *G*_*ss*_) has been taken out and the eigenvalues of ${\bar{G}}_{ss}$ are roughly below the damping factor *α* = 0.85. In the end the resulting vector is multiplied with the matrix *G*_*rs*_ which provides one column of *G*_qr_. This procedure has to be repeated for each of the *N*_*r*_ columns but the number *N*_*r*_ is typically rather modest. We also note that the results obtained in [38] show that an approximate relation holds: 1 − λ_*c*_ ≈ *Σ*_*P*_ = ‖*P*_*r*_‖_1_ where *Σ*_*P*_ is the PageRank probability of the global network concentrated on the subset of *N*_*r*_ selected nodes.

The results obtained here and in [38] for the Wikipedia network show that the contribution of *G*_pr_ is dominant in *G*_*R*_ but it is also kind of trivial with nearly identical columns. Therefore the two small contributions of *G*_*rr*_ and *G*_qr_ are indeed very important for the interpretation even though they only contribute weakly to the overall column sum normalization.

The meaning of *G*_*rr*_ is rather clear since is gives direct links between the selected nodes. In contrast, the meaning of *G*_qr_ is significantly more interesting since it generates indirect links between the *N*_*r*_ nodes due to their interactions with the global network environment. We note that *G*_qr_ is composed of two parts *G*_qr_ = *G*_qrd_ + *G*_qrnd_ where the first diagonal term *G*_qrd_ represents a probability to stay on the same node during multiple iterations of ${\bar{G}}_{ss}$ in (12) while the second nondiagonal term *G*_qrnd_ represents indirect (hidden) links between the *N*_*r*_ nodes appearing due via the global network. We note that in principle certain matrix elements of *G*_qr_ can be negative, which is possible due to negative terms in $Q_{c}=1-P_{c}$ appearing in (12). However, for all subsets considered in this work the total weight of negative elements was negligibly small (at most some 10^−3^) of the total weight 1 for *G*_*R*_).

It is convenient to characterize the strength of 3 components in (10) by their respective weights *W*_*rr*_, *W*_pr_, *W*_qr_ given respectively by the sum of all matrix elements of *G*_*rr*_, *G*_pr_, *G*_qr_ divided by *N*_*r*_. By definition we have *W*_*rr*_ + *W*_pr_ + *W*_qr_ = 1. All numerical data of the reduced Google matrix of groups of proteins considered here are publicly available at the web site [46].

### Global network reconstruction for GM12878 and K562 cell lines

The transcriptional networks for normal GM12878 and cancer K562 cell lines were obtained from the web-site http://encodenets.gersteinlab.org/ (files enets7.K562_proximal_filtered_network.txt, enets8.GM_proximal_filtered_network.txt) accompanying the original publication [10]. SIGNOR network for *H.Sapiens* was downloaded from the SIGNOR web-site http://signor.uniroma2.it/downloads.php. Both transcriptional and SIGNOR networks were represented as simple interaction format (SIF) files and merged by simple concatenation. They were further processed in in Cytoscape [58] with use of BiNoM plugin [12, 59] for finding shortest and second shortest paths, and copy-paste operations.

Merged transcriptional and signaling networks are provided in S1 and S2 Files and in [46].

### Definitions of functionally related groups of proteins

The composition of AKT-mTOR pathway and the set of direct transcriptional targets of E2F1 protein were downloaded from the Atlas of Cancer Signaling Network (ACSN) database [47], by using GMT files of version 1.1 available from the ACSN web-site http://acsn.curie.fr (sets E2F1-TARGETS and AKT-mTOR gene sets).

The group of proteins related to proliferation was determined as a set of 50 gene names top-contributing to the transcriptomic signature associated to the cell cycle through a large-scale pan-cancer analysis of transcriptomic data [49].

All protein lists are provided in the S1 File and in [46].

### Performance and method implementation

Due to analytical derivation of the reduced Google matrix, the application of the formalism to the networks with several thousands or even tens of thousands of nodes takes only seconds on a standard computer (Intel(R) Core(TM) i7-6600U CPU @ 2.60GHz—8Gb RAM). The code for the method is highly optimized to be used with much larger networks. For computational experiments in this study, we used FORTRAN-based implementation of the method. In the nearest future, we plan to develop a Cytoscape plugin performing the reduced Google matrix computation, which will require re-implementing the method in Java.

The results of PageRank and CheiRank calculations are provided in S3 File. The results of reduced Google matrix method application are provided in S4 File.

## Discussion

The results of application of high-throughput technologies in modern molecular biology are more and more frequently presented in the form of complex networks, representing measured causal relations between biological molecules. For example, systematic application of Chip-Seq technology for a significant number of transcription factors can result in the global cell line-specific reconstruction of transcriptional networks [10]. Despite many methods aimed at the analysis of complex networks, there is still a need for mathematically rigorous and computationally efficient methods able to quantify complex non-local network topologies, especially in the case of directed networks.

In this work we show that the global Google matrix and the reduced Google matrix approaches represent useful tools for the analysis of directed interaction networks in biology.

We show that the global analysis of a directed biological network using Google matrix and by computing node PageRanks and CheiRanks and their relative changes in cancer allows obtaining insights about specific and precise aspects of how the biological network topology evolve in different biological contexts.

PageRank and CheiRank quantify the network structure taking into account more complex non-local network patterns comparing to widely used local network topology indices such as connectivity degree or network clustering coefficient. As it has been shown in this paper, high PageRank might correspond to a particular role of a protein in the regulatory network, such as accumulating multiple incoming regulations downstream of a long cascade of molecular interactions or triggering an avalanching signal propagation upstream of multiple hub regulators. These roles can not be easily captured by the standard network analysis methods.

The reduced Google matrix approach is a novel method in the analysis of biological networks which allows quantifying indirect (hidden) connections between members of a specified subset of network nodes. These connections represent paths of oriented graph edges through the global network and involving nodes outside the specified set. This approach is applied to the global network of directed protein-protein interactions, with a focus on some groups of proteins corresponding to a well-defined biological function (cell survival signaling, cell proliferation), obtained by different methods (prior knowledge-based or by data-driven approaches). We show that application of the reduced Google matrix approach leads to inferring a meaningful set of indirect interactions highlighting existence of specific biological programs not reflected in the structure of direct relations between the members of a protein set. We also show that the structure of such hidden relations can be modified from one condition to another, reflecting some global changes in the wiring of, for example, global transcriptional networks during cancer or differentiation.

In this study, we investigated the effect of rewiring of the slow transcriptional regulation network onto the global connectivity of the network, assuming that the structure of the signaling network changes relatively little. Of course, this is a strong simplifying assumption. In reality, cancer mutations can affect important network hubs in the signaling network, and the signaling network structure might also significantly change. However, estimating the functional impact from a mutation in a gene onto the function of a protein in a regulatory network remains a difficult challenge. Our methodology can be further adapted for the joint analysis of the high-throughput omics data (such as mutation data) and the network structure.

Stated in a general way, the reduced Google matrix approach suggests an exact analytical formula for quantifying at once all the functional pair-wise proximities for a set of entities in a global directed biological network. The previously developed approaches are more simplistic in this respect and usually based on quantification of the shortest and the second shortest oriented paths in the network, which does not allow quantifying universally multiple possible ways to wire the connections. For example, quantifying multiple overlapping shortest paths of the same length connecting two proteins in a network, requires ad-hoc scoring strategies and frequently neglects all possibilities of by-passing or complex feedback loops in the path distribution. Moreover, path-based approaches for quantifying the functional distance between many protein pairs easily become computationally inefficient for large networks. Here, we suggest an analytical formalism which is computationally efficient for the typical sizes of the biological networks involving tens of thousands of nodes and hundreds of thousands of interactions.

A promising application of the reduced Google matrix is in mathematical modeling of molecular pathways [60–63]. Construction of a mathematical pathway model usually starts with defining a restrictive set of biological molecules or processes most closely related to the studied phenomenon. Currently, the number of such model elements (proteins) can not be very large. Therefore, there is always a danger of neglecting important indirect causal relations between the elements via regulations passing through the global network in which a given pathway is embedded. The reduced Google matrix method allows systematically inferring indirect regulations, in a context-specific manner, as it is demonstrated in the current study.

Google matrix, or Googlomics, methodology can be used in other types of directed networks appearing in biology such as state transition graphs resulting from the analysis of Boolean models of pathways [64].

Overall, the developed methodology allows combining global structural analysis of large biological networks characterized by context-specific and dynamical re-wiring together with the focused analysis of specified biological processes, without neglecting the role of the global context.

## Supporting information

S1 File Input network files used for the analysis. Merged transcriptional and signaling networks are provided in Simple Interaction Format, and the lists of functionally related genes are provided as plain text files.
(ZIP)

S2 File Cytoscape sessions containing the results of network analysis and visualization. (ZIP)

S3 File Excel file containing PageRank and CheiRank calculations and their analysis. (XLSX)

S4 File Excel file containing the results of reduced Google matrix method application. (XLSX)

S1 Fig Correlation analysis between pairs of comparable centrality measures. The analysis is performed for a directed graph representing the SIGNOR signaling network. INDEG, OUTDE, PAGER, CHEIR, INCLO, OUTCL, BETWE, AUTHO, HUBS signify indegree, outdegree, PageRank, CheiRank, in-closeness, out-closeness, betweenness, authorities and hubs centrality measures respectively. The numbers on top of the plot are Spearmann correlation coefficients. Simple regression lines are shown in each plot.
(TIF)
