# Supplementary figures and images for: Inferring hidden causal relations between pathway members using reduced Google matrix of directed biological networks
Source: PLoS One. 2018 Jan 25;13(1):e0190812. doi: 10.1371/journal.pone.0190812 (PMC5784915; doi:10.1371/journal.pone.0190812)

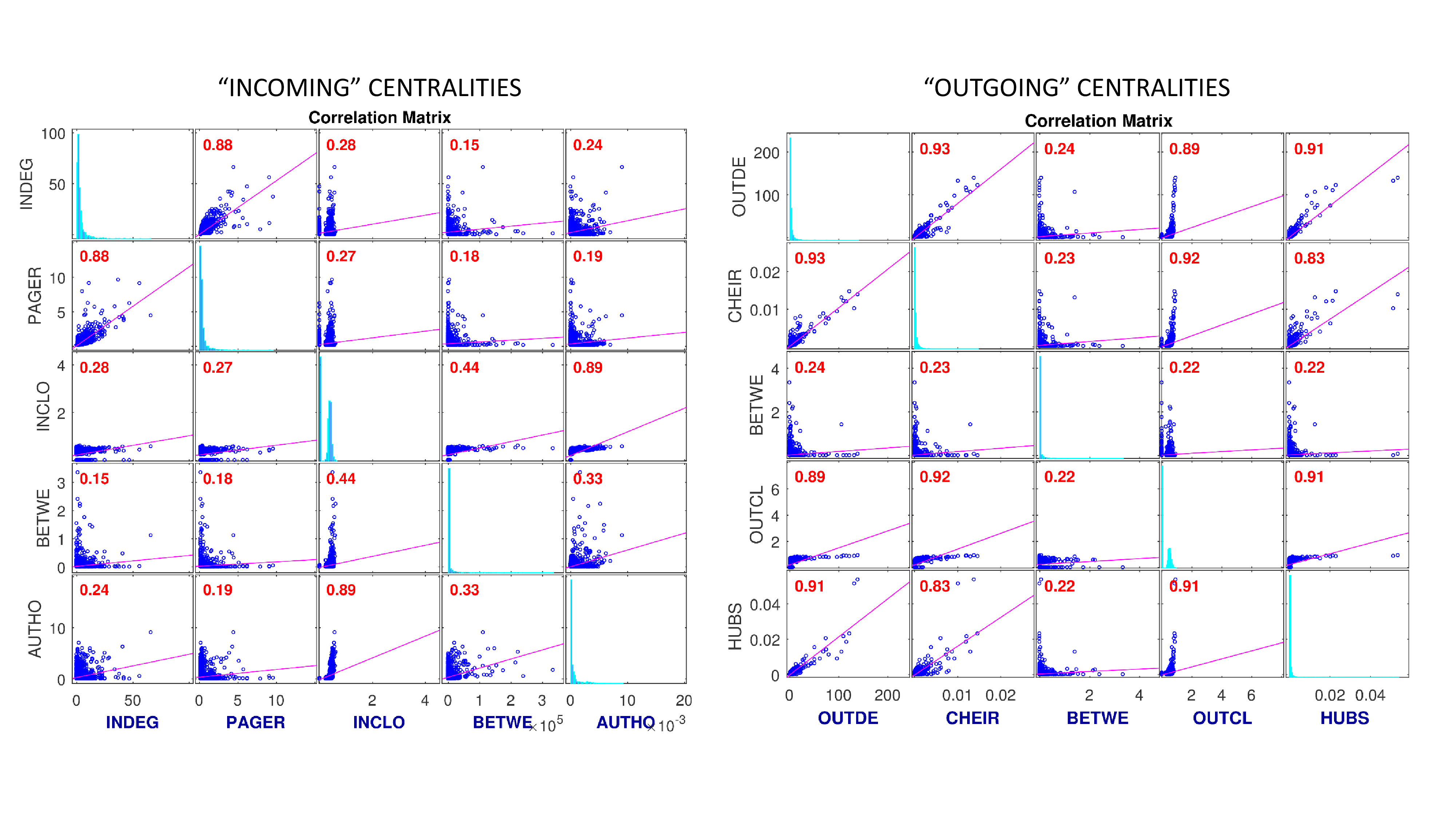

Supplement: S1 Fig — The analysis is performed for a directed graph representing the SIGNOR signaling network. INDEG, OUTDE, PAGER, CHEIR, INCLO, OUTCL, BETWE, AUTHO, HUBS signify indegree, outdegree, PageRank, CheiRank, in-closeness, out-closeness, betweenness, authorities and hubs centrality measures respectively. The numbers on top of the plot are Spearmann correlation coefficients. Simple regression lines are shown in each plot. (TIF) [file pone.0190812.s005.tif]
